# Evaluation of a rapid diagnostic test (CareStart™ Malaria HRP-2/pLDH (Pf/pan) Combo Test) for the diagnosis of malaria in a reference setting

**DOI:** 10.1186/1475-2875-9-171

**Published:** 2010-06-18

**Authors:** Jessica Maltha, Philippe Gillet, Emmanuel Bottieau, Lieselotte Cnops, Marjan van Esbroeck, Jan Jacobs

**Affiliations:** 1 Department of Medical Microbiology, Faculty of Health, Medicine and Life Sciences (FHML), Maastricht, The Netherlands; 2 Department of Clinical Sciences, Institute of Tropical Medicine (ITM), Antwerp, Belgium

## Abstract

**Background:**

Malaria Rapid Diagnostic Tests (RDTs) are widely used for diagnosing malaria. The present retrospective study evaluated the CareStart™ Malaria HRP-2/pLDH (Pf/pan) Combo Test targeting the *Plasmodium falciparum *specific antigen histidine-rich protein (HRP-2) and the pan-*Plasmodium *antigen lactate dehydrogenase (pLDH) in a reference setting.

**Methods:**

The CareStart™ Malaria HRP-2/pLDH (Pf/pan) Combo Test was evaluated on a collection of samples obtained in returned international travellers using microscopy corrected by PCR as the reference method. Included were *P. falciparum *(n = 320), *Plasmodium vivax *(n = 76), *Plasmodium ovale *(n = 76), *Plasmodium malariae *(n = 23) and *Plasmodium *negative samples (n = 95).

**Results:**

Overall sensitivity for the detection of *P. falciparum *was 88.8%, increasing to 94.3% and 99.3% at parasite densities above 100 and 1,000/μl respectively. For *P. vivax, P. ovale *and *P. malariae*, overall sensitivities were 77.6%, 18.4% and 30.4% respectively. For *P. vivax *sensitivity reached 90.2% for parasite densities above 500/μl. Incorrect species identification occurred in 11/495 samples (2.2%), including 8/320 (2.5%) *P. falciparum *samples which generated only the pan-pLDH line. For *P. falciparum *samples, 205/284 (72.2%) HRP-2 test lines had strong or medium line intensities, while for all species the pan-pLDH lines were less intense, especially in the case of *P. ovale*. Agreement between observers was excellent (kappa values > 0.81 for positive and negative readings) and test results were reproducible. The test was easy to perform with good clearing of the background.

**Conclusion:**

The CareStart™ Malaria HRP-2/pLDH (Pf/pan) Combo Test performed well for the detection of *P. falciparum *and *P. vivax*, but sensitivities for *P. ovale *and *P. malariae *were poor.

## Background

With an estimated 247 million cases yearly, malaria is one of the most prevalent infectious diseases, killing 881,000 persons annually [1]. Early diagnosis and treatment are necessary to prevent severe malaria and death. Microscopy still is the reference method for diagnosis, though expertise may be lacking in both endemic and non-endemic settings [2]. Malaria Rapid Diagnostic Tests (RDTs) were introduced in the nineties and have undergone many improvements. Initially two-band tests were used, consisting of a control line and a *P. falciparum *specific test line, either histidine-rich protein-2 (HRP-2) or *P. falciparum *specific lactate dehydrogenase (Pf-pLDH). The later developed three-band RDTs detect both a *P. falciparum *specific antigen and an antigen of the four *Plasmodium *species, either aldolase or pan *Plasmodium*-specific pLDH (pan-pLDH) [2]. By now more than 60 RDT brands and over 200 different products have been developed. Of those, the WHO and Foundation for Innovative New Diagnostics (FIND) evaluated 70 from 26 manufacturers [3,4]. Of these products, 39 are three-band tests that detect and differentiate *P. falciparum *from non-*falciparum *species. The CareStart™ Malaria HRP-2/pLDH (Pf/pan) Combo Test is a three-band RDT detecting HRP-2 and pan-pLDH. The aim of the present study was to evaluate its performance in reference conditions when challenged against a collection of stored clinical samples comprising the four *Plasmodium *species.

## Methods

### Study design

The CareStart™ Malaria HRP-2/pLDH (Pf/pan) Combo Test was retrospectively evaluated in a reference laboratory on a panel of stored blood samples obtained in international travellers suspected of malaria. The reference method was microscopy corrected by Polymerase Chain Reaction (PCR). The study design was in compliance with the STARD guidelines for presentation of diagnostic studies [5].

### Patients and Materials

A panel was selected from a collection of EDTA-blood samples stored at -70°C at the Institute of Tropical Medicine (ITM), Antwerp, Belgium. Between January 1996 and May 2009 these samples were obtained in patients suspected of malaria, including international travellers and, to a lesser extent, natives of endemic countries returning from visiting friends and relatives. Most of these samples were obtained in patients attending the outpatient clinic of the ITM, another part was sent by other Belgian laboratories to ITM for confirmation in the scope of the national reference laboratory. The samples collected at ITM were aliquoted and frozen at -70°C the day of collection. Between collection and storage, the samples remained a maximum of 8 hours at laboratory temperature (below 25°C). The samples submitted by Belgian laboratories for confirmation, were sent by mail and had been exposed to ambient temperature for the period of shipment (generally 24 hours, with a maximum of 48 hours). The delay and processing before storage at -70°C had been validated and were compliant with routine laboratory procedures. The selected panel included samples with the four malaria species at varying parasite densities as well as malaria-negative samples. The samples were classified in regions according to the United Nations classification of geographical region and composition [6].

### Reference Method

All samples were blindly analyzed by microscopy and real-time polymerase chain reaction for diagnosis of malaria, species identification and determination of parasite density, as described previously [7]. A species-specific PCR was adapted from Rougemont et al as described previously [7,8]. In case of discordant results between microscopy and PCR, the result of PCR was used as the reference method.

### Test platforms

The CareStart™ Malaria HRP-2/pLDH (Pf/pan) Combo Test is a lateral flow antigen detection test in a cassette format. It is a three-band RDT targeting HRP-2 and pan-pLDH. The presence of a unique HRP-2 line indicates an infection with *P*. *falciparum*, whereas a unique pan-pLDH line is found in infection with one or more of the non-*falciparum *species. The presence of both HRP-2 and pan-pLDH lines indicates an infection with *P*. *falciparum *or a mixed infection with *P*. *falciparum *and one or more of the non-*falciparum *species.

For the evaluation of CareStart™ Malaria HRP-2/pLDH (Pf/pan) Combo Test, test kits from four different lot numbers were used for evaluation, supplied in two kit presentations : a self-test kit containing 60 individually wrapped packages including cassette, lancet, assay buffer, sample pipette and instructions, and a kit designed for laboratory use with 25 individually packed cassettes. The test kits had been stored between 18°C and 24°C before use. All malaria tests carried out at ITM are accredited in accordance with the requirements of the standard NBN EN ISO 15189:2007.

### Test procedures

Tests were performed according to the instructions of the manufacturer, except that the plastic transfer straws supplied in the kit were replaced by a transfer pipette (Finnpipette, Helsinki, Finland). Readings were carried out at daylight assisted by a standard electric bulb by three subsequent observers, of whom the first always was the one performing the tests. The first observer performed readings at 20 minutes (which is the reading time recommended by the manufacturer), followed by observers 2 and 3 within an additional 10 minutes. The observers were blinded to each other's readings and to the results of microscopy and PCR.

In case no control line appeared the test was considered invalid and was repeated. To score line intensities we used a scoring system of five categories as defined previously [7]: none (no line visible), faint (barely visible line), weak (paler than the control line), medium (equal to the control line) or strong (stronger than the control line). The test results were based on consensus agreement, which means that an identical result read by at least two out of three observers was withheld. In case of no consensus, the results of the first reader were considered.

To assess inter-observer agreement, results of positive and negative readings as well as line intensities were considered. Test reproducibility was evaluated by testing 15 samples representing all species at variable parasite densities on five consecutive occasions.

### Statistical Analysis

Sensitivity and specificity were calculated separately for *P. falciparum *and the non-*falciparum *species with 95% confidence intervals (C.I.). The interpretation of test results for *P. falciparum *and the non-*falciparum *species is shown in Table 1. Samples with pure gametocytaemia were included among the *P. falciparum *species. The Pearson Chi-square test was used to determine significance of results, or in case of small sample size, a two-tailed Fisher's exact test. A p-value < 0.05 was considered as significant.

**Table 1:** Interpretation of test results

For *P. falciparum*		
Test Line(s) visible	Species identification by microscopy corrected by PCR	
	
	*P. falciparum*	*P. vivax, P. ovale, P. Malariae */no parasites detected

Only HRP-2 or both HRP-2 and pan-pLDH	True positive	Species mismatch*/false positive
No test line or only pan-pLDH	False negative/species mismatch**	True negative

**For non-*falciparum *species**		

Test Line(s) visible	Species identification by microscopy corrected by PCR	
	
	*P. vivax*, *P. ovale *or *P. malariae*	*P. falciparum */no parasites detected

Only pan-pLDH	True positive	Species mismatch*/false positive
No test line or only HRP-2 or both HRP-2 and pan-pLDH	False negative/species mismatch***	True negative

* Non-*falciparum *species diagnosed as *P. falciparum*

** *P. falciparum *diagnosed as non-*falciparum *species

*** Non-*falciparum *species diagnosed as *P. falciparum *or as a mixed infection with *P. falciparum*

Inter-observer agreements for line intensities and positive and negative test results were expressed by kappa values for each pair of observers and by the percentage of overall agreement between the three observers. To assess strength of associations between line intensity readings and parasite densities Cramer's V for categorical variables was used [7]. To assess the potential interference of sample storage on test sensitivity, multivariate analysis (logistic regression) was used for each *Plasmodium *species separately. It was hypothesized that sensitivity declined in relation to (i) decreasing parasite densities (ii) longer duration of storage at -70°C and (iii) that there was an interaction between the parasite densities and the duration of storage (antigen degradation related to the amount of antigen present at the beginning). For the multivariate analysis, parasite densities were included after log10 transformation and declines of sensitivity were expressed as Odds ratios. Analyses were conducted using Stata11 (Stata Corporation, Texas, USA).

### Analysis of species mismatch

In case of species mismatch (*P. falciparum *diagnosed as non-*falciparum*, or non-*falciparum *diagnosed as *P*. *falciparum*), the sample was retested with the CareStart™ Malaria HRP-2/pLDH (Pf/pan) Combo Test and with two other RDTs (based on HRP-2 and HRP-2/pan-pLDH).

### Ease of use

The technicians who performed the tests were asked to evaluate the kit's content and instructions for clarity, and problems and incidents during test performance were consequently recorded.

### Ethical review

The study was approved by the Institutional Review Board of ITM and by the Ethical Committee of Antwerp University, Belgium.

## Results

### Sample collection

The samples were selected from the ITM collection, which consists of 1,200 samples. Mixed infections were excluded and only the first sample of each patient was considered. For *P. falciparum*, which is the most frequently retrieved species at ITM, a panel representing the different parasite densities, stages (including only gametocytes) and geographical origin, was selected. The final panel consisted of 590 samples including infections with *P. falciparum *(n = 320), *Plasmodium vivax *(n = 76), *Plasmodium ovale *(n = 76), *Plasmodium malariae *(n = 23) and *Plasmodium *negative samples (n = 95) (Table 2). These samples were obtained in 590 patients with a male:female ratio of 2.16:1. The median age was 35 years (range 1 - 84 years), only 15 patients (2.5%) were children under the age of five years. Table 3 shows the results of the microscopic identification corrected by PCR: of the 76 *P. ovale *samples, seven (9.2%) had originally been diagnosed as *P. vivax *by microscopy, and four out of 76 (5.3%) *P. vivax *samples had originally been diagnosed as *P. ovale*. The geographical distribution of the samples used is shown in Table 2. Samples were obtained in Africa (n = 444), Asia (n = 54), Latin America and the Caribbean (n = 12) and Oceania (n = 4). Of 76 samples no data on the geographic origin were known and could not be retrieved. Most (268/320, 83.8%) *P. falciparum *infections had been acquired in sub-Saharan Africa.

**Table 2:** Species distribution and geographical origin of the 590 samples used for evaluation of the CareStartTM Malaria HRP-2/pLDH (Pf/pan) Combo Test

		Species identification by microscopy corrected by PCR				
		
Geography		P. falciparum	P. vivax	P. ovale	P. malariae	No parasites seen
						
**Africa (n = 444)**	Eastern Africa	18	12	9	5	10
	Northern Africa	1		1		1
	Middle Africa	97	5	24	7	37
	Southern Africa	6	1		1	1
	Western Africa	145	4	27	6	25
	Africa	1				
						
**Asia (n = 54)**	Eastern Asia		1			3
	Southern Asia	4	16	1		5
	South-Eastern Asia	10	10			1
	Western Asia	1				1
	Asia					1
						
**Latin America** **and the Caribbean (n = 12)**	Caribbean			1		1
	South America		6	1		3
						
**Oceania** **(n = 4)**	Melanesia	1	2			
	Micronesia		1			
						
**No data (n = 76)**		36	18	12	4	6
						
**Total (n = 590)**		320	76	76	23	95

**Table 3:** Samples tested: results of species identification by microscopy versus PCR

	Results corrected by PCR					
		
Results of microscopy	Negative	*P. falciparum*	*P. vivax*	*P. ovale*	*P. malariae*	Total
Negative	95					95
*P. falciparum*		320				320
*P. vivax*			72	7		79
*P. ovale*			4	69		73
*P. malariae*					23	23
**Total**	95	320	76	76	23	590

### Test characteristics

No invalid test results were observed. One cassette was broken, and one blister contained no cassette. Table 4 shows the number of positive HRP-2 and pan-pLDH lines for all species. Tables 5 and 6 show the test characteristics according to parasite density. For *P. falciparum*, the overall sensitivity was 88.8%. Sensitivity was related to parasite density: it was 69.6% at parasite densities below 100/μl and increased to 94.3% and 99.3% at parasite densities above 100 and 1,000/μl respectively. Nineteen out of 28 false-negative samples had a parasite density below 100/μl (including five samples with pure gametocytaemia), eight had parasite densities ranging from 120 - 467/μl and the remaining sample had a parasite density of 703/μl. All of these infections had been acquired in sub-Saharan Africa. Eight (2.5%) *P. falciparum *samples only reacted with the pan-pLDH line and consequently represented species mismatch, as they were incorrectly diagnosed as non-*falciparum *species. Parasite densities of these samples ranged from 32 - 371/μl, except for one sample with a parasite density of 1,123/μl; all had been acquired in sub-Saharan Africa (Tables 3, 4 and 6). The species mismatch results were reproducible upon retesting with CareStart™ Malaria HRP-2/pLDH (Pf/pan) Combo Test. Additional testing of these samples with two other RDT brands (HRP-2 and HRP-2/pan-pLDH) scored five of them as *P. falciparum; *the three other samples, obtained in travellers returning from Ghana and DRC, were negative (parasite densities were 32, 70 and 210/μl).

**Table 4:** Test line results of the CareStart™ Malaria HRP-2/pLDH (Pf/pan) Combo Test for all samples (n = 590)

	HRP-2 line positive		HRP-2 line negative	
Samples	pan-pLDH line positive	pan-pLDH line negative	pan-pLDH line positive	pan-pLDH line negative
*P. falciparum *(n = 320)	244	40	8*	28
*P. vivax *(n = 76)	2*	-	59	15
*P. ovale *(n = 76)	-	1*	14	61
*P. malariae *(n = 23)	-	-	7	16
Negative (n = 95)	-	-	-	95

* Species mismatch

**Table 5:** Sensitivities and specificities of CareStart™ Malaria HRP-2/pLDH (Pf/pan) Combo Test for the detection of *P. falciparum*

Results of microscopy corrected by PCR	Numbers	Identified as *P. falciparum *by CareStart™ Malaria HRP-2/pLDH (Pf/pan) Combo Test	% Sensitivity (95% C.I.)	% Specificity (95% C.I.)
**All *P. falciparum *samples**	320	284	88.8 (84.8-92.0)	
Pure gametocytaemia	17	12	70.6 (44.0-89.7)	
Asexual parasite density 0-100/μl	56	39	69.6 (55.9-81.2)	
Asexual parasite density 101-200/μl	35	29	82.9 (66.4-93.4)	
Asexual parasite density 201-1,000/μl	79	72	91.1 (82.6-96.4)	
Asexual parasite density > 1,000/μl	133	132	99.3 (95.9-100)	
Asexual parasite density > 100/μl	247	233	94.3 (90.7-96.9)	
				
**All other species and no parasites seen**	270	3		98.9 (96.8-99.8)
No parasites seen	95	0		100.0 (96.2-100)
*P. vivax*	76	2		
*P. ovale*	76	1		
*P. malariae*	23	0		

**Table 6:** Sensitivities and specificities of CareStart™ Malaria HRP-2/pLDH (Pf/pan) Combo Test for the detection of non-*falciparum *species

Results of microscopy corrected by PCR	Numbers	Identified as non-*falciparum *species by CareStart™ Malaria HRP-2/pLDH (Pf/pan) Combo Test	% Sensitivity (95% C.I.)	% Specificity (95% C.I.)
**All non-*falciparum *species combined**	175	80	45.7 (38.2-53.4)	
				
***P. vivax *samples, total**	76	59	77.6 (66.6-86.4)	
				
Parasite density ≤ 500/μl	25	13	52.0 (31.3-72.2)	
Parasite density > 500/μl	51	46	90.2 (78.6-96.7)	
				
***P. ovale *samples, total**	76	14	18.4 (10.5-29.0)	
Parasite density ≤ 500/μl	35	1	2.9 (0.1-14.9)	
Parasite density > 500/μl	41	13	31.7 (18.1-48.1)	
				
***P. malariae *samples, total**	23	7	30.4 (13.2-52.9)	
Parasite density ≤ 500/μl	11	1	9.1 (0.2-41.3)	
Parasite density > 500/μl	12	6	50.0 (21.1-78.9)	
				
***P. falciparum *and negative samples, total**	415	8		98.1 (96.2-99.2)
*P. falciparum*	320	8		97.5 (95.1-98.9)
No parasites seen	95	0		100.0 (96.2-100)

For the non-*falciparum *species, there were 80/175 (45.7%) samples correctly identified, two samples had both a visible HRP-2 and pan-pLDH line, a single sample showed a unique HRP-2 line and 91 samples showed no test line (Table 4). Species mismatch results were reproducible upon retesting with the CareStart™ Malaria HRP-2/pLDH (Pf/pan) Combo Test: upon testing with two other RDT brands they were diagnosed as non-*falciparum *species. Overall sensitivities for *P. vivax, P. ovale *and *P. malariae *were 77.6%, 18.4% and 30.4% respectively (Table 6). None of the *Plasmodium *negative samples was scored as positive.

### Line intensity reading

Tables 7 and 8 list the line intensity readings related to parasite density. For *P. falciparum*, the majority (205/284, 72.2%) of positive HRP-2 test lines was read as strong or medium, and 27.8% (79/284) were read as weak or faint. Pan-pLDH test lines were read as strong and medium in 129 (51.4%) and as weak or faint in 122 (48.6%) of 251 positive test lines. For the pan-pLDH line intensity in non-*falciparum *species, there was a clear difference between the three species: the *P. vivax *samples generated strong and medium line intensities in half (30/61, 49.2%) of positive samples, whereas *P. ovale *uniquely showed weak or faint lines. For *P. malariae*, 3 out of 7 positive pan-pLDH lines were read as medium line intensity.

**Table 7:** Line intensity consensus reading for the CareStart™ Malaria HRP-2/pLDH (Pf/pan) Combo Test for HRP-2 according to parasite densities

	**Line intensity readings, number of samples**					
		
Results of microscopy corrected by PCR	Negative	Faint	Weak	Medium	Strong	Total
**All *P. falciparum *samples**	36	13	66	66	139	320
Pure gametocytaemia	5	3	3	2	4	17
Asexual parasite density 0-100/μl	17	5	19	8	7	56
Asexual parasite density 101-200/μl	6	2	11	9	7	35
Asexual parasite density 201-1,000/μl	7	3	13	18	38	79
Asexual parasite density > 1,000/μl	1		20	29	83	133
Asexual parasite density > 100/μl	14	5	44	56	128	247
						
**All other species and no parasites seen**	267	2	1			270
No parasites seen	95					95
*P. vivax*	74	1	1			76
*P. ovale*	75	1				76
*P. malariae*	23					23

**Table 8:** Line intensity consensus reading for the CareStart™ Malaria HRP-2/pLDH (Pf/pan) Combo Test for pan-pLDH according to parasite densities

	Line intensity readings, number of samples					
		
Results of microscopy corrected by PCR	Negative	Faint	Weak	Medium	Strong	Total
**All *P. falciparum *samples**	69	60	62	12	117	320
Pure gametocytaemia	8	2	4		3	17
Asexual parasite density 0-100/μl	36	17	3			56
Asexual parasite density 101-200/μl	13	11	10	1		35
Asexual parasite density 201-1,000/μl	11	30	34	3	1	79
Asexual parasite density > 1,000/μl	1	0	11	8	113	133
Asexual parasite density > 100/μl	25	41	55	12	114	247
						
**All other species and no parasites seen**	188	20	29	14	19	270
No parasites seen	95					95
*P. vivax*	15	7	24	11	19	76
*P. ovale*	62	11	3			76
*P. malariae*	16	2	2	3		23

For both the HRP-2 and the pan-pLDH test lines, there was a significant relation between line intensity and parasite density with a substantial correlation (HRP-2: V = 0.353, p < 0.001; pan-pLDH: V = 0.590, p < 0.001), but there was considerable overlap between categories. For *P. falciparum*, faint HRP-2 lines occurred exclusively at parasite densities below 1,000/μl, whereas a strong HRP-2 line intensity indicated a parasite density higher than 100/μl in a vast majority of samples (128/139, 92.1%). In addition a pan-pLDH line of strong intensity indicated in all but four (114/117, 96.6%) *P. falciparum *samples a parasite density exceeding 1,000/μl. Of the latter four samples one had a parasite density of 930/μl and the three other samples had pure gametocytaemia. In case of species mismatch for both *P. falciparum *and non-*falciparum *species, line intensity readings were faint or weak for the discordant test lines.

### Inter-observer agreement and reproducibility

For both HRP-2 and pan-pLDH lines, overall agreement and kappa values between pairs of observers for positive and negative readings were excellent, for line intensities they were good (Table 9). Most differences in line intensity readings between two observers occurred within one category of difference (218/231 (94.4%) and 288/294 (98.0%) for HRP-2 and pan-pLDH respectively).

**Table 9:** Overall agreement and inter-observer agreement between pairs of observers for the CareStart™ Malaria HRP-2/pLDH (Pf/pan) Combo Test assay

	**Overall agreement between three observers (%)**	**Agreement between pairs of observers (kappa values)**
	
Results expressed as positive and negative readings		
		
HRP-2	98.0	0.98, 0.97, 0.97
pan-pLDH	90.3	0.87, 0.87, 0.87
		
Results expressed as line intensity readings		
		
HRP-2	80.7	0.82, 0.80, 0.79
pan-pLDH	75.3	0.77, 0.77, 0.77

**Figure 1:**
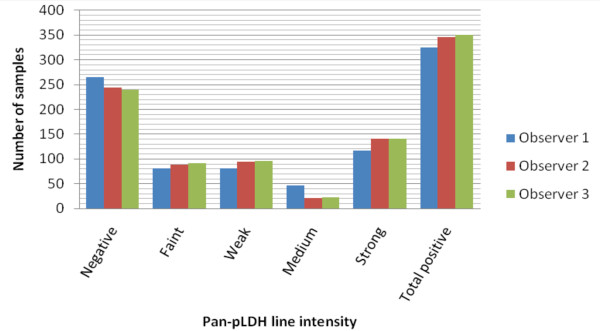
**Pan-pLDH line intensity readings per observer**.

The first observer (who read the test exactly within the recommended reading time of 20 minutes) tended to score weaker pan-pLDH test lines and less positive results compared to observer 2 and 3 (Figure 1), although the differences did not reach statistical significance. As a result, sensitivities for non-*falciparum *species were slightly higher for observers 2 and 3 (49.1% and 48.6%) compared to observer 1 (44.6%). On the other hand, observer 2 and 3 scored, on different occasions, two times a faint positive pan-pLDH test line in case of a negative sample. For HRP-2 no such tendencies in differences were observed.

Tables 10 and 11 list the results of reproducibility for HRP-2 and pan-pLDH. The results for HRP-2 and pan-pLDH lines were reproducible. For the HRP-2 lines, all differences occurred within a single category of intensity. In two samples this resulted in subsequent faint and negative readings. For the pan-pLDH lines, differences occurred within one (n = 5) and two (n = 2) line intensity categories, and two samples showed subsequent faint and negative readings.

**Table 10:** Reproducibility of the CareStart™ Malaria HRP-2/pLDH (Pf/pan) Combo Test: HRP-2 line intensities

	Readings HRP-2 line Observer		
	
Species and parasite density	Observer 1	Observer 2	Observer 3
***P. falciparum***			
85 asexual parasites/μl	S S S S S	S S S S S	S S S S S
			
90 asexual parasites/μl	M M M M M	M W M M W	M M M M M
			
180 asexual parasites/μl	N N F N N	N N F N N	N N N N N
			
200 asexual parasites/μl	M M M M M	W M W M W	W M M M M
			
244 asexual parasites/μl	M M M M M	M M M W M	M M M M M
			
465 asexual parasites/μl	M M S M M	M M S M M	M S S S S
			
720 asexual parasites/μl	S M S S S	M M M S S	S M S S S
			
4,464 asexual parasites/μl	M M M M M	M M M M M	S M M S S
			
1,000,000 asexual parasites/μl	W W W M W	W W W W F	W W W W W
			
***P. malariae***			
275 asexual parasites/μl	N F N N N	N F N N N	N F N N N
			
1,215 asexual parasites/μl	N N N N N	N N N N N	N N N N N
			
***P. ovale***			
468 asexual parasites/μl	N N N N N	N N N N N	N N N N N
			
1,100 asexual parasites/μl	N N N N N	N N N N N	N N N N N
			
***P. vivax***			
432 asexual parasites/μl	N N N N N	N N N N N	N N N N N
			
846 asexual parasites/μl	N N N N N	N N N N N	N N N N N

HRP-2 line intensity of the test performed on five consecutive moments.

Each character represents a distinct test. N = None, F = Faint, W = Weak, M = Medium, S = Strong

**Table 11:** Reproducibility of the CareStart™ Malaria HRP-2/pLDH (Pf/pan) Combo Test: pan-pLDH line intensities

	Readings pan-pLDH line Observer		
	
Sample and parasite density	Observer 1	Observer 2	Observer 3
***P. falciparum***			
85 asexual parasites/μl	N N N N N	N N N N N	N N N N N
			
90 asexual parasites/μl	F F F F F	F F F F F	N F F F F
			
180 asexual parasites/μl	F F F F F	F F F F F	F F F F F
			
200 asexual parasites/μl	W W W W W	W W F W F	F W W F F
			
244 asexual parasites/μl	N N N N N	N N N N N	N N N N N
			
465 asexual parasites/μl	F F W W F	F F F F F	F F F F W
			
720 asexual parasites/μl	W W W W W	W F W W F	W W W W F
			
4,464 asexual parasites/μl	W W W W W	W W F W W	W W F W W
			
1,000,000 asexual parasites/μl	S S S S S	S S S S S	S S S S S
			
***P. malariae***			
275 asexual parasites/μl	N N N N N	N N N N N	N N N N N
			
1,215 asexual parasites/μl	W W W W W	F W F F F	N W F F W
			
***P. ovale***			
468 asexual parasites/μl	N N N N N	N N N N N	N N N N N
			
1,100 asexual parasites/μl	N N N N N	N N N N N	N N N N N
			
***P. vivax***			
432 asexual parasites/μl	W W W W W	W W W W W	W W W W W
			
846 asexual parasites/μl	M M M M M	M S W W W	M M W W M
			

pan-pLDH line intensity of the test performed on five consecutive moments.

Each character represents a distinct test. N = None, F = Faint, W = Weak, M = Medium, S = Strong

### Effect of parasite density and duration of storage on test sensitivity

As suspected by the sensitivities shown in Tables 5 and 6, the relation between parasite densities and test sensitivities was statistically significant in a multivariate model for all four *Plasmodium *species (Table 12). In the same model, an effect of storage, *i.e*. lower sensitivities for samples stored for long versus short periods, was noted only for *P. ovale*. If only samples stored from 2001 onwards were considered, sensitivity for *P. ovale *(n = 38) was 29.0% (95% C.I. 15.4% - 45.9%).

**Table 12:** Effect of duration of storage and parasite density on the sensitivity for each *Plasmodium *species, multivariate analysis

	Odds Ratio	P	95% CI
	
***P. falciparum *(n = 320)**			
Number of years of storage	0.97	0.675	0.86-1.10
Log10 parasite densities	2.04	0.000	1.50-2.76
***P. vivax *(n = 76)**			
Number of years of storage	0.94	0.451	0.79-1.11
Log10 parasite densities	3.56	0.003	1.55-8.18
***P. ovale *(n = 76)**			
Number of years of storage	0.73	0.003	0.60-0.90
Log10 parasite densities	12.19	0.005	2.12-70.23
***P. malariae *(n = 23)**			
Number of years of storage	0.80	0.185	0.57-1.12
Log10 parasite densities	33.10	0.029	1.43-768.88

The decline in sensitivity in relation to low parasite density and long duration of storage was expressed by Odds ratios.

### Ease of use

The CareStart™ Malaria HRP-2/pLDH (Pf/pan) Combo Test was scored as easy to perform. The clearing of the test strip was good, and the test lines were easy visualized. The HRP-2 and pan-pLDH test lines showed slightly different color tones (more blue) as compared to the control line. The test instructions in the package insert were scored as clear and the use of pictures was well appreciated. There were slight discordances in interpretation of the test results between the instructions of the self-test kits and the laboratory kits. For instance, the former stated that only the absence of both the control line and the test lines should be read as invalid, whereas the instructions of the laboratory test mentioned the absence of the control line by itself as an invalid test. In addition, the self-test mentioned a positive HRP-2 and pan-pLDH line as positive for *P. falciparum*, whereas the laboratory test added the option of mixed infection with *P. falciparum *as well. Finally, the unique presence of a pan-pLDH test line should be considered as a *P. vivax *infection according to the instructions of the self-test kit, whereas the laboratory test more correctly mentioned the possibility of (mixed) infections by the three non-*falciparum *species.

## Discussion

In this study, the performance of the CareStart™ Malaria HRP-2/pLDH (Pf/pan) Combo Test was retrospectively evaluated on a large panel (n = 590) of samples obtained in returned travellers suspected of malaria. Overall sensitivity for the detection of *P. falciparum *was 88.8%, reaching 94.3% and 99.3% in samples with parasite densities above 100/μl and 1,000/μl respectively. Overall sensitivities for *P. vivax, P. ovale *and *P. malariae *were 77.6%, 18.4% and 30.4% respectively, reaching 90.2%, 31.7% and 50.0% for parasite densities above 500/μl. No positive results occurred among the *Plasmodium *negative samples; species mismatches occurred in 2.2% (11/495) of samples. Agreement between observers was good and test results were reproducible. The test was easy to perform with good clearing of the background. Test line intensities for the HRP-2 line were strong and medium in the majority of cases, but line intensities for the pan-pLDH lines were lower, especially in the case of *P. ovale*.

The evaluation of an RDT in a reference setting is a logical step preceding evaluations in field trials [9], but this retrospective approach has its limitations [7,10]. For instance, it was not possible to explore discordant or unexpected test results by reviewing patient files or by testing for interferences such as the rheumatoid factor. In addition, test conditions in the reference setting are more favourable compared to the field setting. Further, an influence of sample storage was apparent for *P. ovale*, resulting in a possible underestimation of the actual sensitivity. For the other species, no such effect was noted, and a previous prospective study showed no effect of storage on the stability of the HRP-2 antigen [11]. Finally, stringent criteria were used for defining test characteristics: the eight *P. falciparum *samples that showed only a pan-pLDH line were scored as "species mismatches", as this result represents a major diagnostic error, *i.e*. the misdiagnosis of a *P. falciparum *infection as a non-*falciparum *infection. However, these results could also be considered as a correct diagnosis of "malaria", thereby increasing the overall sensitivity up to 91.3%.

The CareStart™ Malaria HRP-2/pLDH (Pf/pan) Combo Test has recently been evaluated as part of the WHO/FIND Malaria RDT Evaluation Program [3]. This evaluation showed for *P. falciparum *and *P. vivax *detection rates (percentage of naturally infected diluted human samples detected by the product) of respectively 97.5% and 90.0% at parasite densities of 200/μl, and detection rates of 100.0% and 95.0% at parasite densities of 2,000-5,000/μl [3].

The detection rates as demonstrated by WHO/FIND are slightly higher as compared to the sensitivities found in the present study, but the differences were not statistically significant. Comparisons are difficult as the present study included unprocessed clinical samples with a wild range of parasite densities and the WHO/FIND evaluation used series of diluted samples at fixed parasite densities (200/μl and 2,000-5,000/μl).

A similar product of the same company, the CareStart™ Pf/Pv Combo (detecting HRP-2 and *P. vivax-*specific pLDH) has been evaluated in Ethiopia [12]. This study reported higher sensitivities for *P. falciparum *(99.4%). However, it included exclusively *P. falciparum *samples with parasite densities above 100/μl, which is above the detection threshold of most RDTs. In addition, small differences may also be explained by the fact that we included *P. falciparum *samples with pure gametocytaemia as positive samples: in the scope of travel medicine this is a reasonable option [13], but in the present study five out of 17 samples did not react with the HRP-2 line and hence decreased overall sensitivity. Another product of the same company, the CareStart™ Malaria pLDH (Pf/pan) Combo Test has been evaluated in a field study in Madagascar [14]. This study reported sensitivities for *P. falciparum *that are comparable to the present one, including low values at parasite densities < 100/μl (60,0%) and increasing sensitivity at higher parasite densities (100% at > 500/μl). For *P. vivax *there were only nine samples included, making comparison difficult. The same product (CareStart™ Malaria pLDH (Pf/pan) Combo Test) and another product of the CareStart™ brand (Malaria pLDH (pan)) have recently been evaluated in Myanmar [15]. Reported sensitivities for the detection of *P. vivax *were significantly higher than those found in the present study in case of the CareStart™ Malaria pLDH (pan) (91.0%), but for the CareStart™ Malaria pLDH (pan/Pf) they were in line with the present findings (78.5%). Studies evaluating other RDTs in non-endemic countries report similar sensitivities as those found for the CareStart™ Malaria HRP-2/pLDH (Pf/pan) Combo Test in the present study: for *P. falciparum *they ranged from 87.5-99.0%, with one exception of 76.2% [7,10,16-21]. For *P. vivax*, RDTs detecting pan-pLDH showed sensitivities of 33.5% and 62.0%-95.0%,[7,10,13,18,20,22] compared to 46.0%-93.0% [13] for those RDTs targeting aldolase. The increase of sensitivities at higher parasite densities is a well-known phenomenon with breakpoints around 100/μl (*P. falciparum*) and 500/μl (*P. vivax*) [7,10,13,22,23].

Unlike the WHO/FIND evaluation, the present study included *P. ovale *and *P. malariae *samples, for which sensitivities were poor. Previous studies that included *P. ovale *or *P. malariae *species reported sensitivities for these species combined between 36 - 95% for pan-pLDH and 7 - 80% for aldolase based RDTs [13]. In addition, a previous study with a study design similar to the present one reported sensitivities for *P. ovale *and *P. malariae *of 76.3% and 45.2% respectively [7]. Even taken into account an underestimation of the sensitivity for *P. ovale *(due to an effect of sample storage), the presently found low sensitivities for *P. ovale *and *P. malariae *are of concern given the name and the claimed performance of the test. In a field study however, a related RDT of the same company, the CareStart™ two-band RDT targeting pan-pLDH, detected all six *P. malariae *infections, though parasite densities were not given [24].

Of note, in the present study there were eight *P. falciparum *samples (2.5%) that gave a reaction only with the pan-pLDH line but not with the HRP-2 line. Geographic origin of samples probably did not contribute to the species mismatch, as all eight samples were from patients on their return from sub-Saharan Africa where HRP-2 mutations have not yet been described [25]. Low parasite densities close to the detection threshold may explain for the failure of HRP-2 detection of these samples. This species mismatch is of concern, because misdiagnosis of *P. falciparum *infection and therefore treatment with ineffective drugs can have fatal consequences [26].

In line with studies on other RDTs [7,16,27,28], there was a correlation between line intensities and parasite densities, and there were, although to a lesser extent than observed for another RDT [7], diagnostic clues to parasite densities when scoring line intensities (such as a strong HRP-2 line intensity indicating a parasite density > 100/μl). The low intensities of the pan-pLDH lines, especially for the non-*falciparum *species, are of concern especially when extrapolating the present findings to field settings in both endemic and non-endemic settings, where disregarding faint test lines as negative results is a common mistake [29-33].

The agreement between observers was excellent for positive and negative readings and good for line intensity readings, and test reproducibility was also good. Of interest is the observation that reading beyond the reading time increased the number of positive pan-pLDH lines, with an increase in sensitivity (although not significantly) at the expense of a slightly lower specificity. Antigen-antibody reactions are time dependent, explaining for the increase in sensitivity, but on the other hand, the so-called backflow phenomenon (non-specific binding) may have interfered, explaining for the apparently false-positive reactions observed by one out of three readers in the *Plasmodium *negative samples [34].

The CareStart™ Malaria HRP-2/pLDH (Pf/pan) Combo Test was scored as easy to perform by the present team. The good clearance of the background facilitated reading of faint and weak test lines but the difference in color tone between control and test lines hindered reading of test line intensity. Slight improvements to the package insert and manufacturer's instructions are to be expected, especially with regards to interpretation of the test results. This is of particular concern in the case of the self-test kit, designed for travellers or expatriates, who are among the most inexperienced end-users [19,35].

In conclusion, the present study demonstrated that the CareStart™ Malaria HRP-2/pLDH (Pf/pan) Combo Test performs well for the detection of *P. falciparum *and *P. vivax *infections, but poorly for *P. ovale *and *P. malariae. *The occasional species mismatches, in particular *P. falciparum *samples identified as non-*falciparum *species, are to be explored and improvements in test instructions should be made.

## List of abbreviations

CI: Confidence interval; EDTA: Ethylene diamine tetra-acetic acid; FIND: Foundation for Innovative New Diagnostics; HRP-2: Histidine-rich protein 2; ITM: Institute of Tropical Medicine; pan-pLDH: pan *Plasmodium*-specific parasite lactate dehydrogenase; PCR: Polymerase chain reaction; Pf-pLDH: *Plasmodium falciparum-*specific parasite lactate dehydrogenase; pLDH: *Plasmodium*-specific parasite lactate dehydrogenase; Pv-pLDH: *Plasmodium vivax*-specific parasite lactate dehydrogenase; RDT(s): Rapid diagnostic test(s); STARD: Standards for reporting of diagnostic Accuracy; WHO: World Health Organization.

## Competing interests

The authors declare that they have no competing interests.

## Authors' contributions

JM, PG and JJ designed the study protocol. MvE and EB organized prospective sample collection. JM and PG carried out the test evaluations, LC performed PCR analysis. JM, PG and JJ analyzed and interpreted the results and JM drafted the manuscript. JM performed statistical analysis. All authors read and approved the final manuscript.
